# The prevalence of and potential risk factors for Developmental Language Disorder at 10 years in the Raine Study

**DOI:** 10.1111/jpc.16149

**Published:** 2022-08-03

**Authors:** Samuel D Calder, Christopher G. Brennan‐Jones, Monique Robinson, Andrew Whitehouse, Elizabeth Hill

**Affiliations:** ^1^ School of Allied Health, Faculty of Health Sciences Curtin University Perth Western Australia Australia; ^2^ Health Sciences, College of Health and Medicine University of Tasmania Launceston Tasmania Australia; ^3^ Telethon Kids Institute, The University of Western Australia Perth Western Australia Australia

**Keywords:** Developmental Language Disorder, prevalence, the Raine Study

## Abstract

**Aim:**

This study sought to determine the prevalence of Developmental Language Disorder (DLD) in Australian school‐aged children and associated potential risk factors for DLD at 10 years.

**Methods:**

This study used a cross‐sectional design to estimate the prevalence of DLD in Generation 2 of the prospective Raine Study. Participants included 1626 children aged 10 years with available language data. Primary outcomes included variables matching diagnostic criteria for DLD. Associations of other potential prenatal and environmental variables were analysed as secondary outcomes.

**Results:**

The prevalence of DLD in this sample was 6.4% (*n* = 104) at 10 years. This sub‐cohort comprised 33.7% (*n* = 35) with expressive language deficits, 20.2% (*n* = 21) with receptive language deficits, and 46.2% (*n* = 48) with receptive‐expressive deficits. No significant difference in sex distribution was observed (52.9% male, *p =* 0.799). Children who were exposed to smoke *in utero* at 18 weeks gestation were at increased risk of DLD at 10 years (OR = 2.56, CI = 1.23–5.35, *p =* 0.012).

**Conclusions:**

DLD is a relatively prevalent condition in Australian children, even when assessed in middle childhood years. These findings can inform future research priorities, and public health and educational policy which account for the associations with potential risk factors.

## What is already known on this topic

1. Developmental Language Disorder (DLD) is prevalent and under‐researched.
2. The long‐term impact of DLD is well‐established despite a lack of public awareness.
3. A number of risk factors for DLD have been identified, but more research is needed.

## What this paper adds

1. This study demonstrates that Developmental Language Disorder (DLD) is prevalent at 10 years (6.4%) in a large‐scale Australian prospective birth sample.
2. There are no differences in sex distribution of children with DLD.
3. Maternal smoking during pregnancy is significantly associated with offspring DLD at 10 years.

Developmental Language Disorder (DLD) is a childhood condition that affects the ability to comprehend and use language compared to typically developing peers.^1^ The labels for describing childhood language disorders, which are not associated with another biomedical condition (such as autism or intellectual disability), have evolved over decades, and included specific language impairment^2^ and language disorders of unknown origin.^3^ Poor agreement in terminology for significant early language deficits led to a multinational and multidisciplinary consensus study,^1^ which proposed DLD as the standard label, which aligns with recent updates to international classifications for disability^4^, ^5^ (see Table 1 for a summary of diagnostic criteria).

**Table 1:** Diagnostic criteria for (Developmental) Language Disorder

	DSM‐5 (4): 315.39(F80.9)	ICD‐11 (5): 6A01.2	CATALISE (1)
Persistence	Persistent difficulties in the acquisition and use of language across modalities (i.e., spoken, written, sign language or other) due to deficits in comprehension or production that include the following: Reduced vocabulary (word knowledge and use). Limited sentence structure (ability to put words and word endings together to form sentences based on the rules of grammar and morphology). Impairments in discourse (ability to use vocabulary and connect sentences to explain or describe a topic or series of events or have a conversation).	Persistent deficits in the acquisition, understanding, production or use of language (spoken or signed). The individual's ability to understand, produce or use language is markedly below what would be expected given the individual's age.	Research has shown children are unlikely to catch up to peers spontaneously.
Function	Language abilities are substantially and quantifiably below those expected for age, resulting in functional limitations in effective communication, social participation, academic achievement or occupational performance, individually or in any combination.	Cause significant limitations in the individual's ability to communicate.	Language difficulties that create obstacles to communication or learning in everyday life.
Exclusion	The difficulties are not attributable to hearing or other sensory impairment, motor dysfunction or another medical or neurological condition and are not better explained by intellectual disability (intellectual developmental disorder) or global developmental delay.	The language deficits are not explained by another neurodevelopmental disorder or a sensory impairment or neurological condition, including the effects of brain injury or infection (exclusions: autism spectrum disorder (6A02), disease of the nervous system (8A00‐8E7Z), Deafness not otherwise specified (AB52), selective mutism (6B06)).	Language disorder is not associated with a known biomedical condition (such as: brain injury, acquired epileptic aphasia in childhood, certain neurodegenerative conditions, genetic conditions such as Down Syndrome, cerebral palsy, sensorineural hearing loss, autism spectrum disorder and intellectual disability).
Early onset	Onset of symptoms is in the early developmental period.	Arise during the developmental period, typically during early childhood.	Onset of symptoms is in early childhood, and neurobiological (e.g., male) and environmental (e.g., poverty) risk factors do not preclude a diagnosis

## Prevalence of Developmental Language Disorder

Recent estimates of the prevalence of DLD in English‐speaking countries indicate approximately 7% of 4‐ to 5‐year‐old children meet diagnostic criteria (57–59% male),^2^, ^3^ with a potential reduction in middle childhood (7–9 years).^6^ Estimates are further classified into rates of receptive (comprehension; 54% and 37% at age 7 and 9 years, respectively), expressive (production; 32% and 44% at age 7 and 9 years, respectively), and combined receptive‐expressive language deficits (66% and 62%, at age 7 and 9 years, respectively).^6^ The prevalence of DLD has been estimated in the USA^2^ and the UK^3^ only; each study using different diagnostic criteria. In the USA, researchers identified 7.4% of 5‐year‐old children (*n =* 2009) as meeting diagnostic criteria using a −1.25 standard deviation cut‐off on standardised language tests and a −1.00 standard deviation cut‐off on a test of non‐verbal intelligence (NVIQ). Later, the UK‐based study applied a −1.50 standard deviation cut‐off on standardised language tests and a − 2.00 standard deviation cut‐off on a measure of NVIQ to *n =* 529 children (aged 4‐5‐years) and identified 7.6% to meet the criteria for DLD. Only recently achieving consensus for DLD as a diagnostic label has likely hindered the number of studies evaluating the prevalence of the condition. The outcomes of these studies suggest that roughly two children in every mainstream classroom of 30 students present with a language disorder that is likely to have a persistent and pervasive impact into adulthood.^7^ A history of DLD is associated with lower academic and vocational qualifications.^8^ Males with DLD are four times more likely to engage in anti‐social behaviour^9^ and females with DLD are three times more likely to be victims of sexual abuse.^10^ Children with DLD are an undoubtedly vulnerable population, yet they often go under‐identified and do not receive specialist services.^11^ This is particularly the case for females with DLD who are less likely than males to be referred.^12^

In Australia, much of our awareness of the prevalence and impact of DLD has been informed by international research. To date, there has been no exploration of the prevalence of DLD in Australian children, and few studies have explored current estimates in the middle childhood years internationally. Consequently, there is limited evidence to guide the development of public health and education campaigns specific to the Australian context. Furthermore, evidence of the prevalence of DLD in middle childhood would provide impetus for ongoing support for these children in and beyond the primary school years.

## The Raine Study and risk factors for Developmental Language Disorder

Epidemiological studies are necessary to identify at‐risk populations, investigate risk factors associated with disease and disorders, and to inform public health policy. The Raine Study is a Western Australian prospective pregnancy cohort study that recruited 2900 pregnant women between 1989 and 1991, with 2730 women giving birth to 2868 children between 1989 and 1992.^13^ Mothers and children of the Raine Study have found to be representative of the general population at study follow‐ups.^14^ The large sample size and low selection bias of the Raine Study allow for investigation of prevalence of conditions, as well as the evaluation of relationships between disorders and exposures, such as the potential risk factors shown to be associated with childhood language disorders. These include being male,^15^ preterm birth,^16^ exposure to environmental teratogens *in utero*, such as maternal alcohol and tobacco use,^17^, ^18^ low maternal education,^15^ and low household income.^17^ Other conceptually relevant protective factors, such day‐care attendance, being read to in the early years,^17^ breastfeeding,^19^ and dyadic ‘parentese’^20^ warrant exploration.

## Current study

The aim of the current study was to estimate the prevalence of children meeting diagnostic criteria for DLD at age of 10 years in an Australian population sample. Using the Raine Study data, our first objective was to determine the percentage of children who meet contemporary diagnostic criteria for DLD at age 10. Our second objective was to identify potential risk factors and to assess the magnitude of the relationship between potential risk factors and the presence of DLD at 10 years.

## Methods

### Participants

This study investigated second‐generation (Gen2) Raine Study participants, which included 2868 live births at King Edward Memorial Hospital in Perth, Western Australia between 1989 and 1991. Inclusion criteria for the Raine Study were expecting mothers with a gestational age of 16–20 weeks, English proficiency to communicate with investigators, and residency in Western Australia. Data were analysed from 1‐year (1990–1993), 2‐year (1991–1994), 3‐year (1992–1995) and 10‐year (1999–2002) follow‐ups. At the 10‐year follow‐up, complete language data were available for 1626 Gen2 participants. Cases (*n =* 1240) without complete language data were excluded from this study. Recruitment and follow‐up for the Raine Study were approved by the Human Ethics Committee at King Edward Memorial Hospital. Analysis of existing data was approved by the Raine Study and Curtin Human Research Ethics Committee (HREC approval number: HRE2021‐0117).

### Variables

The primary outcome variable for this study was children meeting diagnostic criteria for DLD at 10 years. This was determined by using scores from the *Clinical Evaluation of Language Fundamentals Third Edition* (CELF‐3)^21^ to measure language functioning and the Raven's Coloured Progressive Matrices (RCPM)^22^ to measure NVIQ. The CELF is a standardised omnibus assessment which is widely used for clinical and research purposes due to its sound psychometric properties.^23^ The CELF‐3 provides an aggregated language index (Total Language Score), a Receptive Language Index, and an Expressive Language Index. All raw scores across primary variables were converted to *z‐*scores for analysis.

Children with DLD were identified according to performance 1.50 standard deviation or below the population mean on language composite scores in the absence of intellectual disability (i.e., NVIQ 2.00 standard deviations or below the population mean), or another biomedical condition that may better explain language difficulties (e.g., autism, intellectual disability or hearing loss). Cases were individually checked for biomedical, or other conditions (parent‐report) and analysis of corresponding ICD‐9 codes. Cases identified to meet the criteria for DLD were also examined to determine if any had been identified to have language disorder diagnosed by a health professional.

Potential risk factors (parent‐report) were collected as secondary outcomes, including: sex, gestational age at birth (<37 weeks), maternal ethnicity, mother spoke a language other than English, maternal education, father's place of residence at birth, household income (<$27 000), smoke and alcohol exposure during pregnancy, history of breastfeeding, day‐care attendance, and if the child was read to at least once per week. We ran *χ* ^2^ tests between children who had and the 1240 cases who did not have language data to test for systematic differences in the distribution on predictive variables. More mothers who did not complete Year 12 provided language data (*χ* ^2^ = 3.94, *P =* 0.048), and more families who had a household income < $27 000 did not provide language data (*χ* ^2^ = 4.06, *P =* 0.044).

### Statistical analysis

Frequency distributions and *χ* ^2^ tests of demographic variables and potential risk factors were analysed based on children meeting diagnostic criteria for DLD, or not, at 10 years. Binomial logistic regressions were used to identify potential risk factors, and to determine the magnitude of the predictive relationship between risk factors and meeting criteria for DLD at age 10. Data were analysed using SPSS version 27.

## Results

The prevalence of participants meeting the criteria for DLD at 10 years was 6.4% (*n* = 104) (Table 2). In the DLD cohort, two cases were identified as having a language disorder by a health professional. Of the total 1626 participants, 1.0% (*n* = 16) of cases were classified as having a language disorder associated with intellectual disability as indicated by NVIQ ≥ −2.00 standard deviations (no corresponding ICD‐9 code provided). The remaining 1506 (92.6%) cases presented without language disorder according to scores on the CELF‐3 and RCPM. The mean chronological age of participants at the 10‐year follow‐up was 10.6 years (SD = 0.2 years, range = 10.2–12.4 years).

**Table 2:** Frequencies of DLD and no DLD and demographic information

	No DLD, *n* (%)	DLD, *n* (%)
Total number	1506 (92.6%)	104 (6.4%)
	16 (1.0%)†	
Socio‐economic status		
<$27 000	835 (54.9%)	56 (53.8%)
≥$27 000	604 (39.7%)	40 (38.5%)
Not stated	83 (5.4%)	8 (7.7%)
Ethnicity		
Caucasian	1345 (88.4%)	95 (91.4%)
Aboriginal	29 (1.9%)	2 (1.9%)
Polynesian	13 (0.8%)	2 (1.9%)
Vietnamese	7 (0.5%)	0
Chinese	68 (4.5%)	2 (1.9%)
Indian	41 (2.7%)	3 (2.9%)
Other	19 (1.2%)	0
Language spoken most at home		
English	1439 (94.5%)	101 (97.1%)
Vietnamese	10 (0.7%)	0
Chinese	18 (1.2%)	0
Italian	1 (0.1%)	0
Greek	3 (0.2%)	0
Spanish	5 (0.3%)	0
Other	46 (3.0%)	3 (2.9%)

DLD, Developmental Language Disorder; NVIQ, non‐verbal intelligence.

† Language disorder associated with intellectual disability (i.e., NVIQ ≥ 2.0 SD below the mean).

Table 3 presents the sex distribution and sub‐classifications of language disorder for the DLD group as determined through Receptive and Expressive Language Indices. There were no sex differences between children with and without DLD. Of the 104 cases with DLD, 33.6% (*n* = 35) presented with expressive language deficits, 20.2% (*n* = 21) presented with receptive language deficits, and 46.2% (*n* = 48) presented with receptive‐expressive language deficits. See Table 4 for *z*‐scores for indices and subtests on the CELF‐3 and the RCPM. On average, the children meeting the criteria for DLD scored ≥2.00 standard deviations below the population mean on the CELF‐3 Total Language Score.

**Table 3:** Prevalence and characteristics of DLD in the Raine Study

Characteristics at 10 years	Male, *n* (%)	Female, *n* (%)	*P* value	Totals, *n* (%)
DLD	55 (52.9%)	49 (47.1%)	0.556	104 (6.4%)
Expressive	18 (51.4%)	17 (48.6%)	0.866	35 (33.7%)
Receptive	9 (42.9%)	12 (57.1%)	0.513	21 (20.2%)
Expressive – receptive	28 (58.3%)	20 (41.7%)	0.248	48 (46.2%)
No DLD	770 (50.6%)	752 (49.4%)	0.626	1522 (93.6%)

DLD, Developmental Language Disorder.

**Table 4:** Participant *z*‐scores on primary variables

	No DLD	DLD
CELF‐3 Total Language Score	0.14	−2.09
CELF‐3 Receptive Language Index	0.12	−1.76
CELF‐3 Expressive Language Index	0.13	−1.83
NVIQ	0.06	−0.86

CELF‐3, Clinical Evaluation of Language Fundamentals Third Edition; DLD, Developmental Language Disorder; NVIQ, non‐verbal intelligence.

Frequency distributions of potential risk factors for DLD at 10 years are presented in Table 5. In the DLD group, there was a higher proportion of children born preterm (*P =* 0.04) and those exposed to smoke *in utero* (*P* = 0.006). Fewer children with DLD at 10 years had their father living at home at birth (*P* = 0.002) and fewer children with DLD were read to at age 3 (*P* = 0.005).

**Table 5:** Frequency distributions of risk factors for DLD

DLD risk variables	No DLD	DLD	*P* value
Sex			
Male	770 (51.1%)	55 (52.9%)	0.651
Female	752 (49.9%)	49 (47.1%)	
Gestational age			
≥37 weeks	1370 (90.0%)	87 (83.7%)	0.040†
<37 weeks	152 (10.0%)	17 (16.3%)	
Ethnicity			
Caucasian	1345 (88.4%)	95 (91.3%)	0.356
Other than Caucasian	177 (11.6%)	9 (8.7%)	
Mother spoke language other than English			
No	1439 (94.6%)	101 (97.1%)	0.257
Yes	83 (5.4%)	3 (2.9%)	
Maternal education (completed Year 10)			
Yes	1379 (90.6%)	89 (85.6%)	0.094
No	143 (9.4%)	15 (14.4%)	
Maternal education (completed Year 12)			
Yes	608 (39.9%)	32 (30.8%)	0.064
No	914 (60.1%)	72 (69.2%)	
Father lived at home at birth			
Yes	1319 (94.3%)	79 (76.0%)	0.002†
No	79 (5.7%)	25 (24.0%)	
Missing	124	0	
Household income			
<$27 000	835 (58.0%)	56 (58.3%)	0.953
≥$27 000	604 (42.0%)	40 (46.7%)	
Missing	83	8	
Early smoke exposure (18 weeks)			
Yes	399 (26.2%)	40 (38.5%)	0.006†
No	1123 (73.8%)	64 (61.5%)	
Alcohol consumed during pregnancy			
Yes	692 (45.5%)	54 (51.9%)	0.205
No	828 (54.5%)	50 (48.1%)	
Missing	2	0	
Breastfeeding			
Breastfed	1132 (89.1%)	79 (89.8%)	0.428
Not breastfed	138 (10.9%)	9 (10.2%)	
Missing	252	16	
Day‐care attendance (2 years)			
Yes	322 (33.2%)	24 (35.3%)	0.727
No	647 (66.8%)	44 (64.7%)	
Missing	553	636	
Day‐care attendance (3 years)			
Yes	532 (47.6%)	42 (53.9%)	0.288
No	585 (52.4%)	36 (46.1%)	
Missing	405	26	
Read to once a week or more (2 years)			
Yes	693 (88.2%)	46 (88.5%)	0.949
No	93 (11.8%)	6 (11.5%)	
Missing	736	52	
Read to once or week or more (3 years)			
Yes	1044 (90.8%)	64 (81.1%)	0.005†
No	106 (9.2%)	15 (18.9%)	
Missing	372	25	

DLD, Developmental Language Disorder.

† *P* value significant at 0.05 threshold.

The logistic regression model was non‐significant, *χ* ^2^(8) = 10.31, *P =* 0.244 (Table 6). The model explained 7.9% of the variance, and correctly identified 94.6% of cases. The children of mothers who reported smoking cigarettes at 18 weeks gestation were at increased risk of DLD at 10 years (*P =* 0.012). Other risk factors that were significant in *χ* ^2^ tests, such as gestational age (*P =* 0.060), father living at home (*P =* 0.319), and being read to at 3 years (*P =* 0.051), were non‐significant predictors. All remaining potential risk factors were also non‐significant.

**Table 6:** Binomial logistic regression of potential risk factors for DLD at 10 years

Risk factors	Odds ratio	95% Confidence interval	*P* value
Sex: male	1.09	0.55–2.13	0.814
Gestational age: <37 weeks	2.36	0.97–5.74	0.060
Ethnicity: other than Caucasian	0.922	0.28–3.01	0.893
Mother spoke language other than English	1.95	0.38–9.98	0.422
Maternal education: did not complete Year 10	0.80	0.25–2.52	0.697
Maternal education: did not complete Year 12	1.67	0.75–3.73	0.212
Father did not live at home	1.63	0.63–4.24	0.319
Household income: <$27 000	0.66	0.30–1.44	0.295
Early smoke exposure at 18 weeks gestation	2.56	1.23–5.35	0.012†
Alcohol consumed during pregnancy	0.81	0.40–1.65	0.562
Did not breastfeed	0.86	0.25–2.98	0.816
Did not attend day‐care at 2 years	1.07	0.50–2.30	0.870
Did not attend day‐care at 3 years	0.68	0.32–1.46	0.326
Read to once a week or more at 2 years: no	0.46	0.17–1.79	0.261
Read to once a week or more at 3 years: no	2.67	0.99–7.15	0.051

DLD, Developmental Language Disorder.

† *P* value significant at 0.05 threshold.

## Discussion

This is the first study to estimate the prevalence of DLD in an Australian cohort aged 10 years. In the Raine Study cohort, prevalence was 6.4%, with 33.7% presenting with expressive language deficits, 20.2% presenting with receptive language deficits, and 46.2% presenting with a combined profile of receptive‐expressive deficits. Our results are consistent with previous studies in English‐speaking countries identifying a prevalence of roughly 7% in early childhood (4–5 years)^2^, ^3^ with a slight decrease in prevalence in middle childhood.^6^ Similarly, receptive‐expressive language deficits comprise the greatest proportion of children meeting diagnostic criteria.^7^ Overall, these estimates suggest that DLD is a relatively stable and common neurodevelopmental disorder. As noted by McGregor (^24^ Table 1, p. 976), DLD is more prevalent than other well‐publicised neurodevelopmental disorders, such as autism (0.65%) and attention‐deficit/hyperactivity disorder (5%).

Previous studies have indicated a marginal difference between males and females meeting the criteria for DLD.^2^, ^3^ A systematic review and meta‐analysis of case history risk factors found being male to be similarly predictive to late talking in identifying language disorder in early childhood.^15^ However, we found no significant differences in the distribution of sex in the unselected Raine Study cohort, and sex was not a significant predictor of meeting criteria for DLD at 10 years. Males have been found to be more likely to be referred for clinical services.^12^ Taken together, these findings speak to referral bias for males and likely under‐detection of females presenting with clinically significant language disorders in childhood. From an access‐to‐service standpoint, educators and health professionals should be vigilant when considering referrals for females who present with potential language and learning challenges. This also highlights the importance of raising public awareness of the presence of DLD in male and female children, as this may inform parents' capacity to identify and advocate for support in females with language and learning challenges.

Our findings provide inconsistent support for risk factors for DLD that have been previously reported.^25^ As DLD is often under‐identified,^11^ perhaps many cases that meet criteria for DLD within the Raine Study cohort were not included in previous analyses.^25^ The current study found that a higher proportion of children meeting the criteria for DLD at 10 years were born preterm, were exposed to smoke *in utero*, had a father that did not live at home, and were read to less than once per week at 3 years. However, the only significant predictor of meeting the criteria for DLD was smoke exposure *in utero*, with the odds of meeting the criteria of DLD 2.56 times greater when mothers smoked at 18 weeks gestation. Smoking during pregnancy has previously been associated with deteriorating and low language outcomes at age 10.^17^ There is a case to be made for exposure to environmental teratogens *in utero*, which may ultimately result in brain‐behaviour differences, and language difficulties in the developing child compared to children whose mothers did not smoke.^26^ In contrast, parental smoking has been argued to be an environmental risk factor rather than a causal risk factor.^18^ That is, although smoking during and after pregnancy was associated with language disorder in 6‐year‐old children, the association was non‐significant once parental education was controlled for, suggesting rather that parental smoking is an indicator of a disadvantaged parenting environment rather than a causal risk factor for language disorder.^18^ Although it is not possible to conclude a causal link between smoke exposure *in utero* and DLD in middle childhood in the current study, this finding reinforces the need for ongoing public awareness of the risks associated with smoking during pregnancy.

### Limitations and future directions

This study leveraged data from the Raine Study cohort, a prospective pregnancy cohort study, to investigate the relationship between early exposures and clinical outcomes with a large unselected and representative Australian population sample. We used data from valid and widely used assessments to determine the prevalence of children who met DLD criteria at 10 years. One key limitation is that significantly fewer participants below the poverty line did not report language outcomes, and so were excluded from the analysis. Therefore, we cannot rule out potential social disadvantage associated with poverty as a significant predictor of DLD. Further, two important criteria for determining a diagnosis for DLD were not addressed. First, the onset of language difficulties in the early years was not considered in this study because there was a high proportion of missing data for cases which would have reduced the statistical power for identifying DLD in middle childhood. Second, the functional impact of DLD was not assessed (e.g., a metric for educational attainment). Another limitation includes the age of the Raine Study data, which began in 1989. Many of the variables explored in the current study were taken from standardised tests which have been superseded by newer editions. This study also did not consider whether plausibly bilingual children (*n* = 3) were tested only on their English language skills. Finally, participants were recruited from only one hospital in Western Australia. Future prospective longitudinal studies should recruit broadly and investigate the comorbidities of literacy and mental health disorders.^7^, ^8^ Follow‐ups should be implemented with frequent intervals using assessment procedures that show utility in diagnostic accuracy in early and middle childhood and functional impact, such as language samples and curriculum‐based assessments.

## Conclusions

The prevalence of DLD in a representative Australian sample of children was 6.4% at 10 years. This is the first study of Australian children estimating the prevalence of DLD, and one of few identifying the proportion of children with a clinically significant language deficit in middle childhood. Despite the high number of potential risk factors included in the Raine Study datasets, smoking while pregnant was the only significant predictor. Of the cohort, many children met the criteria for DLD, the functional impacts of which can persist into adulthood. Early identification of these children using robust tools for measurement is critical. These findings should inform education and public health priorities to raise awareness of DLD and establish the foundation for exploring effective interventions and service delivery models for these at‐risk children.
